# Hyperbaric oxygen therapy promotes consciousness, cognitive function, and prognosis recovery in patients following traumatic brain injury through various pathways

**DOI:** 10.3389/fneur.2022.929386

**Published:** 2022-08-10

**Authors:** Yuwen Chen, Liang Wang, Wenjun You, Fei Huang, Yingzi Jiang, Li Sun, Siye Wang, Su Liu

**Affiliations:** ^1^Department of Rehabilitation Medicine, Affiliated Hospital of Nantong University, Nantong, China; ^2^School of Medicine, Nantong University, Nantong, China; ^3^Department of Rehabilitation, Nantong First People's Hospital, Nantong, China; ^4^Department of Geriatrics, Second Peoples Hospital of Nantong, Affiliated of Nantong University, Nantong, China; ^5^Department of Rehabilitation Medicine, Nantong Health College of Jiangsu Province, Nantong, China

**Keywords:** traumatic brain injury, hyperbaric oxygen therapy, Stockholm CT, quantitative electroencephalography, serum markers, prognosis

## Abstract

**Objective:**

The aim of this study was to investigate the clinical curative effect of hyperbaric oxygen (HBO) treatment and its mechanism in improving dysfunction following traumatic brain injury (TBI).

**Methods:**

Patients were enrolled into control and HBO groups. Glasgow coma scale (GCS) and coma recovery scale-revised (CRS-R) scores were used to measure consciousness; the Rancho Los Amigos scale-revised (RLAS-R) score was used to assess cognitive impairment; the Stockholm computed tomography (CT) score, quantitative electroencephalography (QEEG), and biomarkers, including neuron-specific enolase (NSE), S100 calcium-binding protein beta (S100β), glial fibrillary acidic protein (GFAP), brain-derived neurotrophic factor (BDNF), nerve growth factor (NGF), and vascular endothelial growth factor (VEGF), were used to assess TBI severity. The patients were followed up 6 months after discharge and assessed with the Glasgow outcome scale-extended (GOSE), functional independence measure (FIM), and the disability rating scale (DRS).

**Results:**

The CRS-R scores were higher in the HBO group than the control group at 10 days after treatment. The RLAS-R scores were higher in the HBO group than the control group at 10 and 20 days after treatment. The Stockholm CT scores were significantly lower in the HBO group than the control group at 10 days after treatment. HBO depressed the (δ + θ)/(α + β) ratio (DTABR) of EEG, with lower δ band relative power and higher α band relative power than those in the control group. At 20 days after treatment, the expression of NSE, S100β, and GFAP in the HBO group was lower than that in controls, whereas the expression of BDNF, NGF, and VEGF in the HBO group was higher than that in controls. Six months after discharge, the HBO group had lower DRS scores and higher FIM and GOSE scores than the control group significantly.

**Conclusions:**

HBO may be an effective treatment for patients with TBI to improve consciousness, cognitive function and prognosis through decreasing TBI-induced hematoma volumes, promoting the recovery of EEG rhythm, and modulating the expression of serum NSE, S100β, GFAP, BDNF, NGF, and VEGF.

## Introduction

Traumatic brain injury (TBI) is a public health challenge worldwide with an annual prevalence of approximately 50 million patients with TBI; furthermore, the number of patients with TBI in China exceeds that in most other countries worldwide (1). Many studies have raised public attention regarding damage from TBI with varying severity, which can lead to impaired consciousness, cognitive loss, neurologic deficits, behavioral disturbances, and neuropsychiatric confusion, thus having devastating effects on patients and their families. TBI is expected to cost the global economy approximately 400 billion US dollars every year (2, 3). Approximately 20% cases of TBI are moderate to severe, and 80% cases of TBI are mild; nevertheless, moderate to severe TBI leads to high mortality, disorders of consciousness (DoC), physical and psychosocial deficits, cognitive impairment, and even disability, thus resulting in social and economic burdens (4, 5).

Hyperbaric oxygen (HBO) therapy is defined as the inhalation of 100% oxygen inside a hyperbaric chamber pressurized to >1 atmosphere absolute; this treatment may alleviate secondary injury after TBI through a variety of mechanisms (6). In the past two decades, HBO has been demonstrated to be neuroprotective in patients with TBI through increasing tissue oxygenation, suppressing inflammation, inhibiting apoptosis, decreasing intracranial pressure, and promoting neurogenesis and angiogenesis (7). Moreover, HBO has been found to decrease the incidence of cognitive impairment, improve prognosis, and diminish mortality (8–10).

HBO therapy in TBI has long been controversial, mainly because of the inexact curative effect of HBO treatment, the occurrence of complications including barotraumas, oxygen toxicity, and imperceptible impairments that have not been described in detail, and the lack of clarity regarding the therapeutic mechanism (11–17). In this study, the clinical therapeutic effects of HBO therapy were evaluated on the basis of awareness, cognition, brain imaging and electrophysiology changes, and patient prognosis.

## Materials and methods

### Patients, groups, and ethics

Patients with TBI treated at the Department of Rehabilitation Medicine in the Affiliated Hospital of Nantong University from December 2019 to October 2021 were enrolled and divided into an HBO group and a control group. The inclusion criteria were as follows: (1) The patients had been required to have a prior diagnosis of TBI by the Neurosurgery Department (including traumatic subarachnoid hemorrhage, epidural hematoma, subdural hematoma, intraventricular hemorrhage, diffuse axonal injury, cerebral contusion, and laceration); (2) moderate or severe TBIs due to the injury (a GCS score of 3–8, or post-traumatic amnesia of more than 7 days or loss of consciousness for more than 24 h, is classified as severe TBI; a GCS score of 9–12, or posttraumatic amnesia of 1–7 days, or loss of consciousness between 30 min and 24 h, is classified as moderate TBI) (18); (3) age of 18–80 years. The patients with acute cardiac arrest or hemorrhagic shock at the time of TBI were excluded. Other exclusion criteria for the patients were other major extracranial injuries, infection within the most recent month, previous head trauma, neurological diseases, including ischemic and hemorrhagic stroke, and other prior systemic diseases, including uremia, liver cirrhosis, malignancy, chronic heart disease, and chronic lung disease.

**Figure 1:**
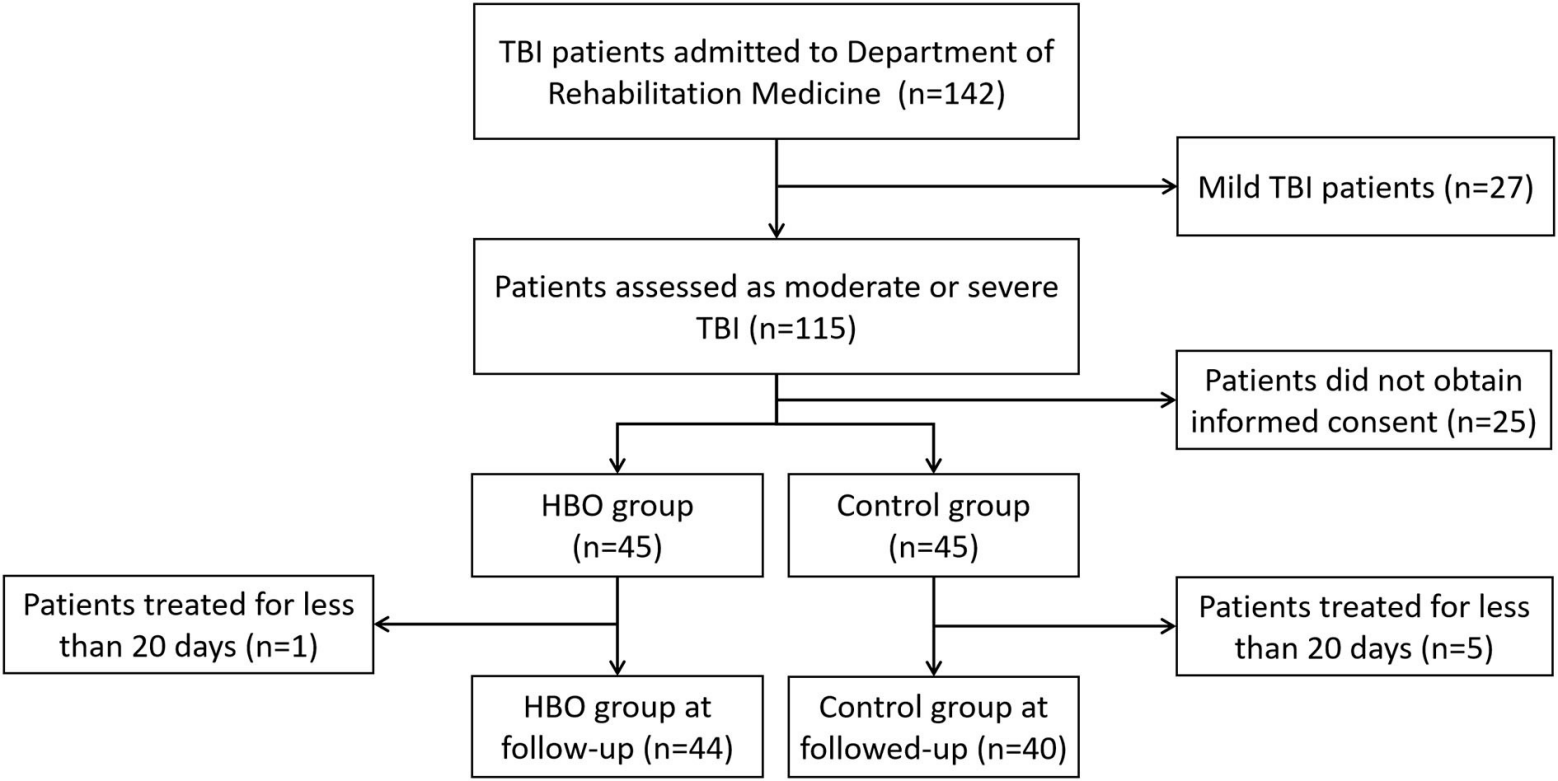
A CONSORT diagram.

The calculated appropriate sample size estimates indicated that 34 patients were required in each group (effect size = 0.8, α = 0.05, power = 0.9). A total of 90 patients with TBI were initially assessed and divided into two groups, and the patients who did not receive HBO treatment because of various human factors, contraindication, or economic reasons were entered the control group; however, one patient lacking information was excluded in the HBO group, and three patients lacking of information and two patients with multiple injuries were excluded in the control group. Eventually, 84 patients were collected: 44 patients in the HBO group and 40 patients in the control group (Figure 1).

This study was approved by the Medical Ethics Committee of the Affiliated Hospital of Nantong University (approval No. 2020-K029), and written informed consent was obtained from all the participants or legal guardians.

### Assessment

#### Neurological function and prognosis assessment

The head trauma severity in both experimental and control groups was assessed with the Glasgow coma scale (GCS) score and the abbreviated injury scale (AIS)-Head score at mission, and state of consciousness was assessed by the coma recovery scale–revised (CRS-R). Cognitive impairment was measured with the Rancho Los Amigos scale-revised (RLAS-R). The patients were assessed by three professionals, who conducted a blind evaluation before the start of rehabilitation instruction after admission, at 3, 10, and 20 days after treatment, by using CRS-R and RLAS-R. After 6 months, the patients were evaluated in a blinded manner with GOSE, DRS, and FIM during the follow-up period.

### CT imaging evaluation and analysis

Routine laboratory examinations were completed after admission and at 3, 10, and 20 days after treatment. To record the Stockholm CT score, professionals first calculated the traumatic subarachnoid hemorrhage (tSAH) score = SAH in convexities (1 if 1–5 mm, 2 if > 5 mm) + SAH in basal cisterns (1 if 1–5 mm, 2 if > 5 mm) + intraventricular hemorrhage (2 if present) (range: 0–6), then calculated the tally score = the midline shift (mm)/10 + the tSAH score/2 – 1 if epidural hemorrhage + 1 if diffuse axonal injury (basal ganglia, splenium or brain stem) + 1 if dual-sided subdural hematoma + 1. All CT scans were acquired in accordance with Neuroradiology Department protocols.

### QEEG evaluation and analysis

The patients had received EEG before treatment and after 20 treatments. Continuous digital EEG data were recorded with the Neurofax EEG-2100 System software, and EEG preprocessing and feature calculation were performed in MATLAB R2019a (MathWorks, Inc., Natick, MA). Gross artifact signals (i.e., those with no identifiable normal EEG activity according to visual inspection) were removed from the unprocessed traces; EEG data segmentation was technically performed in System software on epochs of 2 s with an overlap of 50% (1 s). After fast Fourier transformation, the spectrum was subdivided into frequency bands: δ (0.5–3.5 Hz), θ (4–7.5 Hz), α (8–12.5 Hz), and β (13–30 Hz). The following parameters were calculated: relative power of each frequency band and the (δ + θ)/(α + β) ratio (DTABR).

### Hyperbaric oxygen therapy

The patients started HBO therapy when their vital signs had stabilized after admission, and received a total of 20 treatments continuously in monoplace hyperbaric oxygen chambers (Shanghai 701; Yang Garden Hyperbaric Oxygen Chamber Co., Ltd., Shanghai, China). A chamber pressure of 2.0 atmosphere absolute was chosen, with pressurization for 15 min, oxygen inhalation with constant pressure for 60 min, and decompression for 15 min, according to prior studies (10, 19, 20). During HBO, professional nurses closely monitored the patients and immediately suspended treatment if a serious adverse reaction or an event reflecting intolerance of HBO occurred. Both groups received standardized functional rehabilitation training, routine medical interventions, and nursing care.

### Blood sampling collection and analysis

Venous blood was drawn from the patients before treatment and 3, 10 and 20 days after treatment. Serum was obtained by centrifugation at 1,600 × *g* for 15 min at room temperature, and then stored at −80°C in the Clinical Biobank of Affiliated Hospital of Nantong University until analyses. Serum neuron-specific enolase (NSE), S100 calcium-binding protein beta (S100β), glial fibrillary acidic protein (GFAP), brain-derived neurotrophic factor (BDNF), nerve growth factor (NGF), and vascular endothelial growth factor (VEGF) concentrations were measured separately with ELISA kits according to the manufacturer's instructions (NSE, S100β, GFAP ELISA kits from CUSABIO, Wuhan, China; BDNF, NGF, VEGF ELISA kits from Boster, Wuhan, China).

### Statistical analysis

All statistical calculations were conducted in SPSS ver. 22.0, and graphs were constructed in GraphPad PRISM ver. 8.0. Normally distributed datasets are expressed as the mean ± standard deviation, and skewed datasets are expressed as the median and interquartile range. Categorical variables are reported as counts and proportions. To determine the level of significance between multiple groups, we applied ordinary one-way ANOVA or the Kruskal-Wallis tests as appropriate. Demographic, clinical, and biochemical parameters were compared between experimental and control groups with independent sample *t*-tests or the Wilcoxon–Mann–Whitney U tests as appropriate. Qualitative variables were compared with the Pearson's chi-square test or the Fisher's exact test as appropriate. All statistical tests were two-tailed, and a *p* ≤ 0.05 was considered statistically significant.

## Results

### Patient characteristics

Parameters recorded at admission included age, sex, pupil reaction, systolic arterial pressure, diastolic arterial pressure, and surgery history (Table 1). The mean age of the HBO treatment group was 55.48 ± 15.21 years, and 29 (65.9%) were males. The mean age of the control group was 60.55 ± 9.92 years, and 25 (62.5%) were males. No significant differences were observed between groups regarding time since injury, cause of injury, education, head AIS, and other patient characteristics (*p* > 0.05).

**Table 1:** Patient characteristics.

**Parameter**	**HBO group** **(*n* = 44)**	**Control group** **(*n* = 40)**	***P***
Age, years	55.48 ± 15.21	60.55 ± 9.92	0.077
Gender, *n* (Male/Female)	29/15	25/15	0.745
Time since injury, days	33.09 ± 23.92	35.13 ± 37.64	0.771
Time to start treatment, days (HBOT/rehab)	38.48 ± 15.54	39.90 ± 32.704	0.557
Cause of injury, *n* (%)			0.459
Traffic accidents	28 (63.6)	28 (70.0)	
Fall/Fall from height	10 (22.8)	5 (12.5)	
Others	6 (13.6)	7 (17.5)	
Education, years	5.95 ± 2.12	5.85 ± 2.84	0.848
Head AIS, score	4 (4–5)	4 (3–5)	0.094
GCS, score	7.09 ± 2.96	7.30 ± 2.10	0.708
Hypertension, *n* (%)	10 (22.7)	10 (25.0)	0.807
Diabetes, *n* (%)	3 (6.8)	7 (17.5)	0.182
Unreactive pupils, *n* (%)	13 (29.5)	18 (45.0)	0.143
Received surgery, *n* (%)	29 (65.9)	21 (52.5)	0.211
Tracheostomy, *n* (%)	23 (52.3)	19 (47.5)	0.662
Systolic blood pressure, mmHg^*^	123.95 ± 16.08	125.90 ± 15.52	0.575
Diastolic blood pressure, mmHg^*^	75.25 ± 10.01	78.18 ± 9.55	0.176
Fasting plasma glucose, mmol/L^*^	5.93 ± 1.50	6.29 ± 1.89	0.350

Values are presented as the mean ± standard deviation, median with interquartile range (25th, 75th). HBO, hyperbaric oxygen; AIS, abbreviated injury severity; GCS, Glasgow Coma Scale.

* Systolic blood pressure, diastolic blood pressure and fasting plasma glucose were collected upon admission.

### Effect of HBO therapy on the level of consciousness

**Figure 2:**
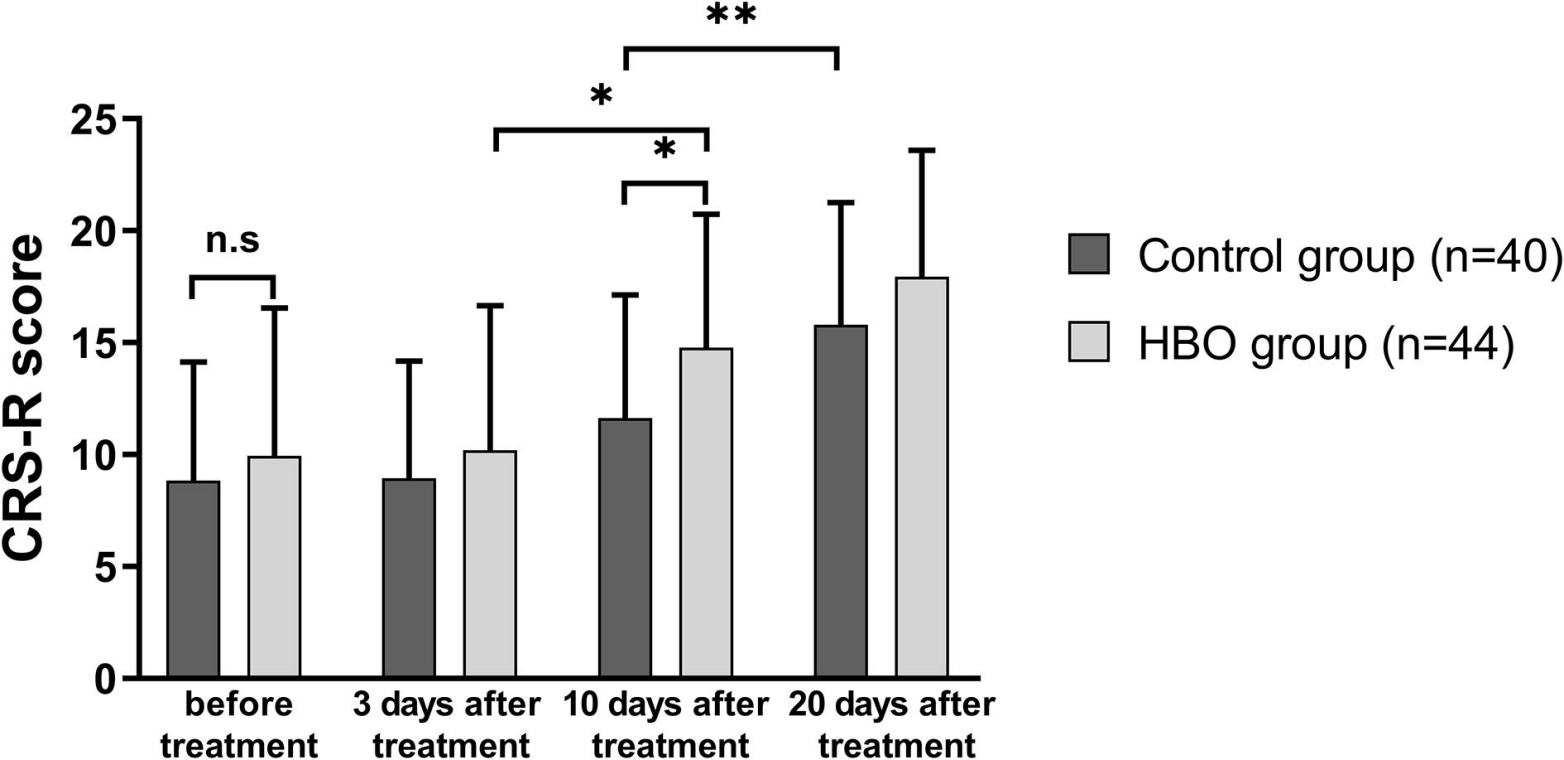
Comparison of the level of consciousness before and after treatment. The HBO group had higher CRS-R scores than the control group at 10 days after treatment (*p* = 0.014). *p < 0.05; **p < 0.01.

The CRS-R scores of the patients in both groups at 20 days after treatment increased with respect to those before treatment. The CRS-R scores in the HBO group were higher than those in the control group at 10 days after treatment (14.77 ± 5.96 vs. 11.63 ± 5.51; *p* = 0.014). The patients with HBO therapy had better recovery of consciousness than those in the control group (Figure 2).

### Effect of HBO therapy on cognitive impairment

**Figure 3:**
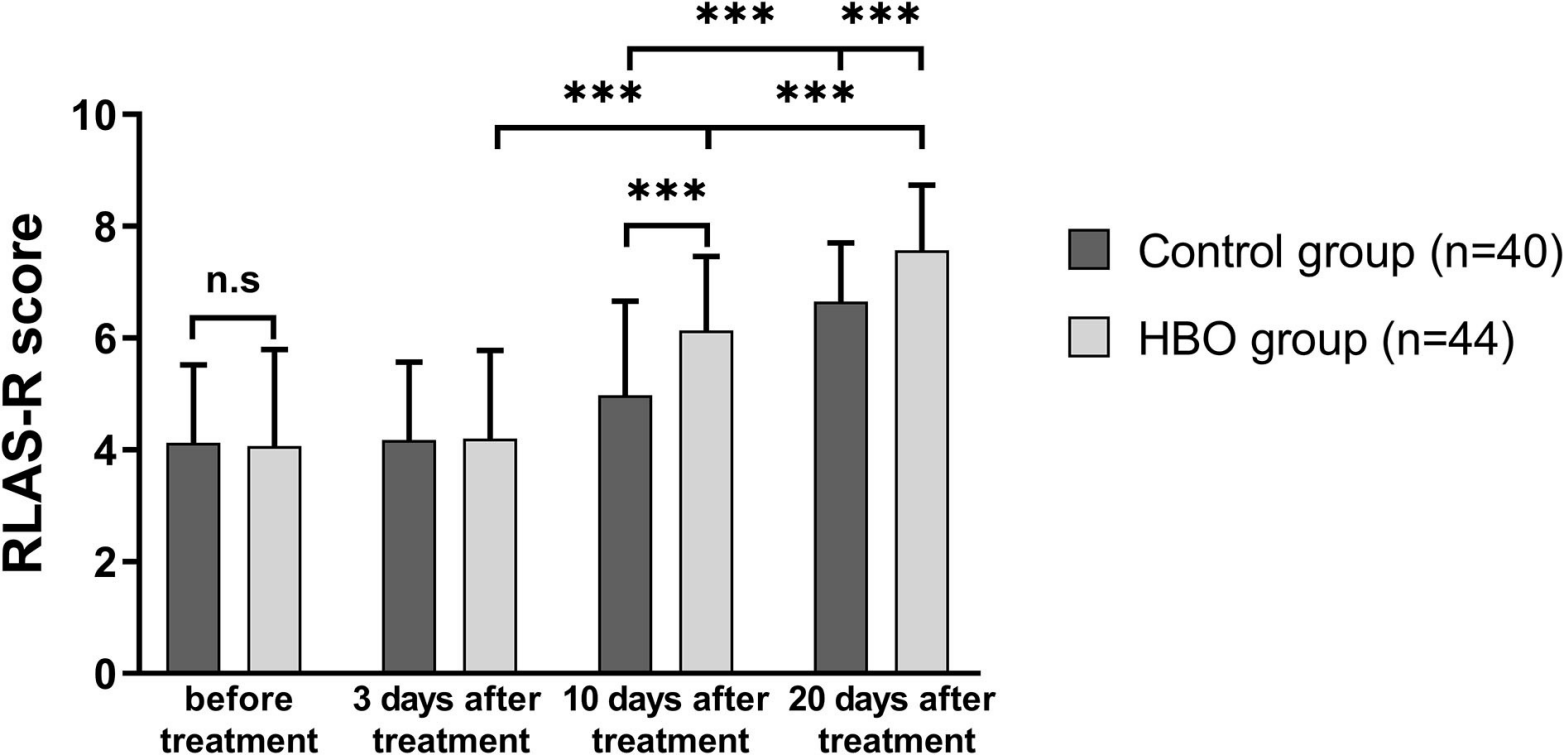
Comparison of RLAS-R scores before and after treatment, showing that the HBO group had higher RLAS-R scores than the control group at 10 days and 20 days after the treatment (*p* = 0.001, *p* < 0.001). ***p < 0.001.

RLAS-R scores of the patients in both groups at 20 days after treatment increased with respect to those before treatment. The RLAS-R scores of the patients with TBI in the HBO group were higher than those in the control group at 10 days (6.14 ± 1.32 vs. 4.98 ± 1.69; *p* = 0.001) and 20 days after treatment (7.57 ± 1.17 vs. 6.65 ± 1.05; *p* < 0.001), thus indicating that the patients with HBO therapy had better cognitive function than those in the control group (Figure 3).

### Effects of HBO therapy on intracranial injuries

**Figure 4:**
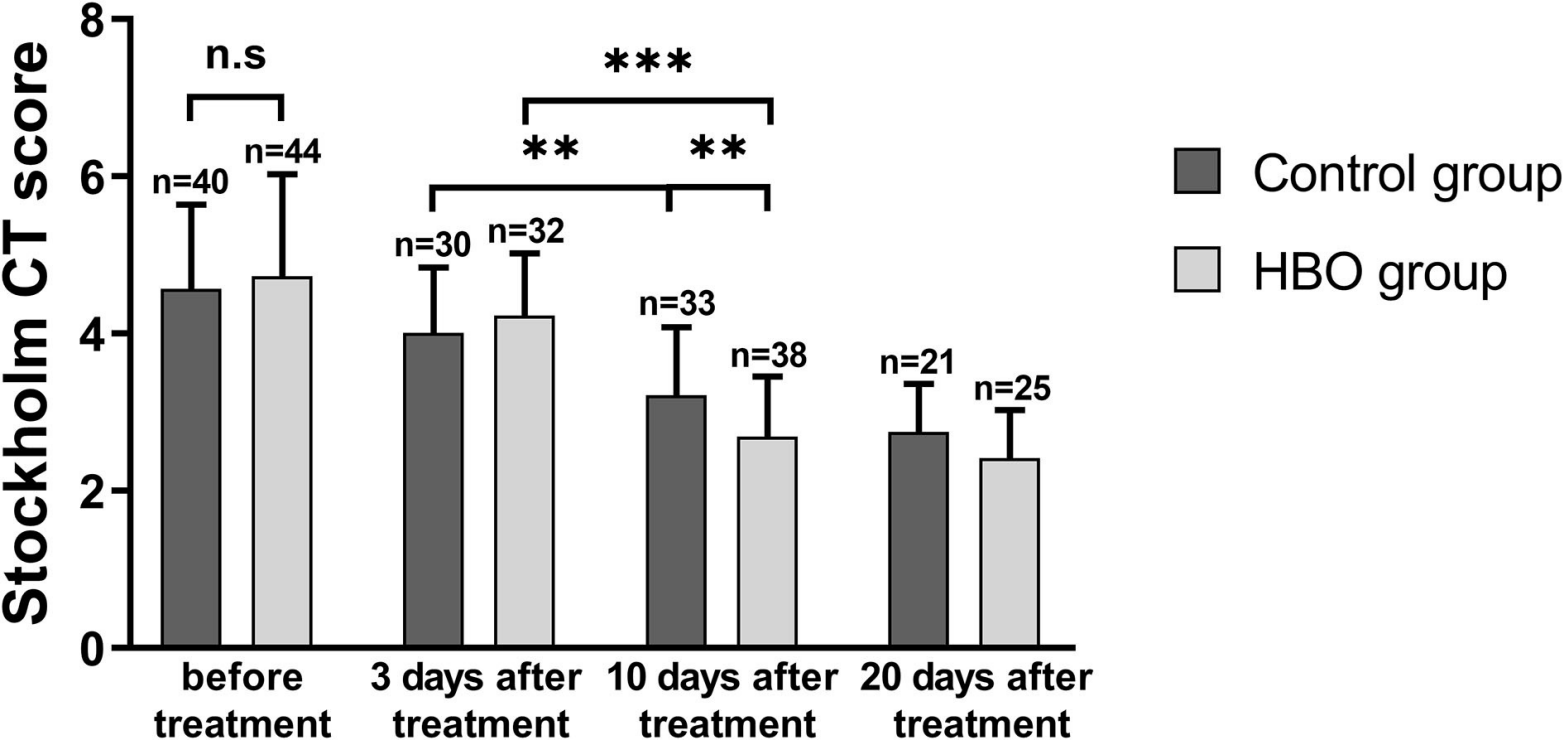
Comparison of Stockholm CT scores before and after the treatment, showing that the HBO group had lower Stockholm CT scores than the control group at 10 days after the treatment (*p* = 0.008). **p < 0.01; ***p < 0.001.

**Figure 5:**
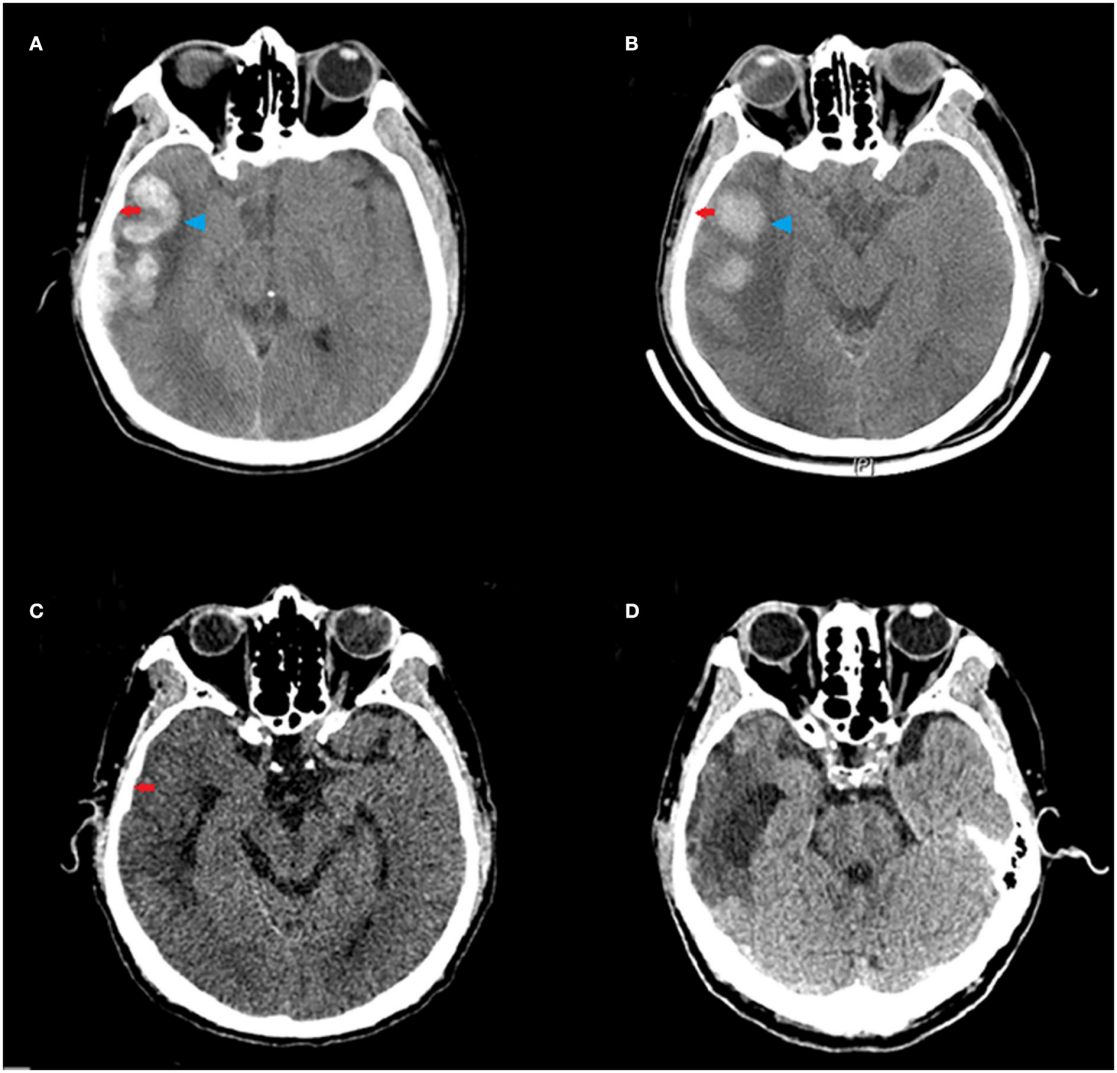
Representative CT scans of a 65-year-old male patient with moderate TBI (GCS = 10) due to a road traffic accident, who underwent HBO therapy. Red arrows indicate subdural hematoma in the right frontal, parietal, temporal, and occipital lobes; blue arrowheads indicate hematoma in the right temporal lobe. **(A)** The CT scan before HBO therapy, showing large hematoma. **(B)** The CT scan at 3 days after the HBO therapy, showing decreased hematoma volume. **(C)** The CT scan at 10 days after the HBO therapy, showing rapid absorption of the hematoma. **(D)** The CT scan at 20 days after the HBO therapy, showing near disappearance of the hematoma.

Stockholm CT scores at 10 days and 20 days after the treatment were lower than those before the treatment. The Stockholm CT scores in the HBO group were lower than those in the control group at 10 days after the treatment (2.69 ± 0.77 vs. 3.22 ± 0.86; *p* = 0.008) (Figure 4). CT scans at different times showed that HBO therapy decreased TBI-induced intracranial hematoma volume (Figure 5).

### Effects of HBO on QEEG relative power and DTABR

No statistical differences in relative powers and DTABRs were observed between the groups before the treatment (*p* > 0.05). Compared with those before the treatment, the relative power of the δ band decreased, and the relative power of the θ and α bands increased in the HBO group, whereas only the relative power of the β band increased significantly in the control group. The relative power of the δ band was lower, and that of the α band was significantly higher in the HBO group than the control group at 20 days after the treatment (*p* = 0.043, *p* = 0.003), but DTABRs showed no statistical differences between the HBO and control groups (*p* = 0.237), thus indicating that HBO might promote the recovery of normal EEG rhythm through decreasing the slow wave fraction of the δ band and increasing the relative power of the α band (Table 2).

**Table 2:** Comparison of relative power and slow wave ratio.

	**HBO group (*****n*** = **15)**		**Control group (*****n*** = **15)**	
	**Before treatment**	**20 days** **after treatment**	**Before treatment**	**20 days** **after treatment**
RP of δ (%)	76.83 ± 3.93	64.47 ± 11.75^a^^b^	73.05 ± 5.93	71.05 ± 6.73
RP of θ (%)	13.59 ± 1.69	16.97 ± 4.92^b^	15.99 ± 2.15	14.90 ± 3.27
RP of α (%)	7.61 ± 2.70	16.83 ± 8.60^a^^b^	8.51 ± 3.79	10.03 ± 2.84
RP of β (%)	1.95 ± 0.45	2.59 ± 4.01	2.44 ± 0.68	4.01 ± 1.28^b^
DTABR	10.19 ± 2.70	5.65 ± 3.12^b^	9.42 ± 3.88	6.87 ± 3.13^b^

HBO, hyperbaric oxygen; RP, relative power; DTABR, (Delta+Theta)/(Alpha+Beta) ratio.

a P < 0.05, vs. control group simultaneously point;

b P < 0.05, vs. before treatment.

**Figure 6:**
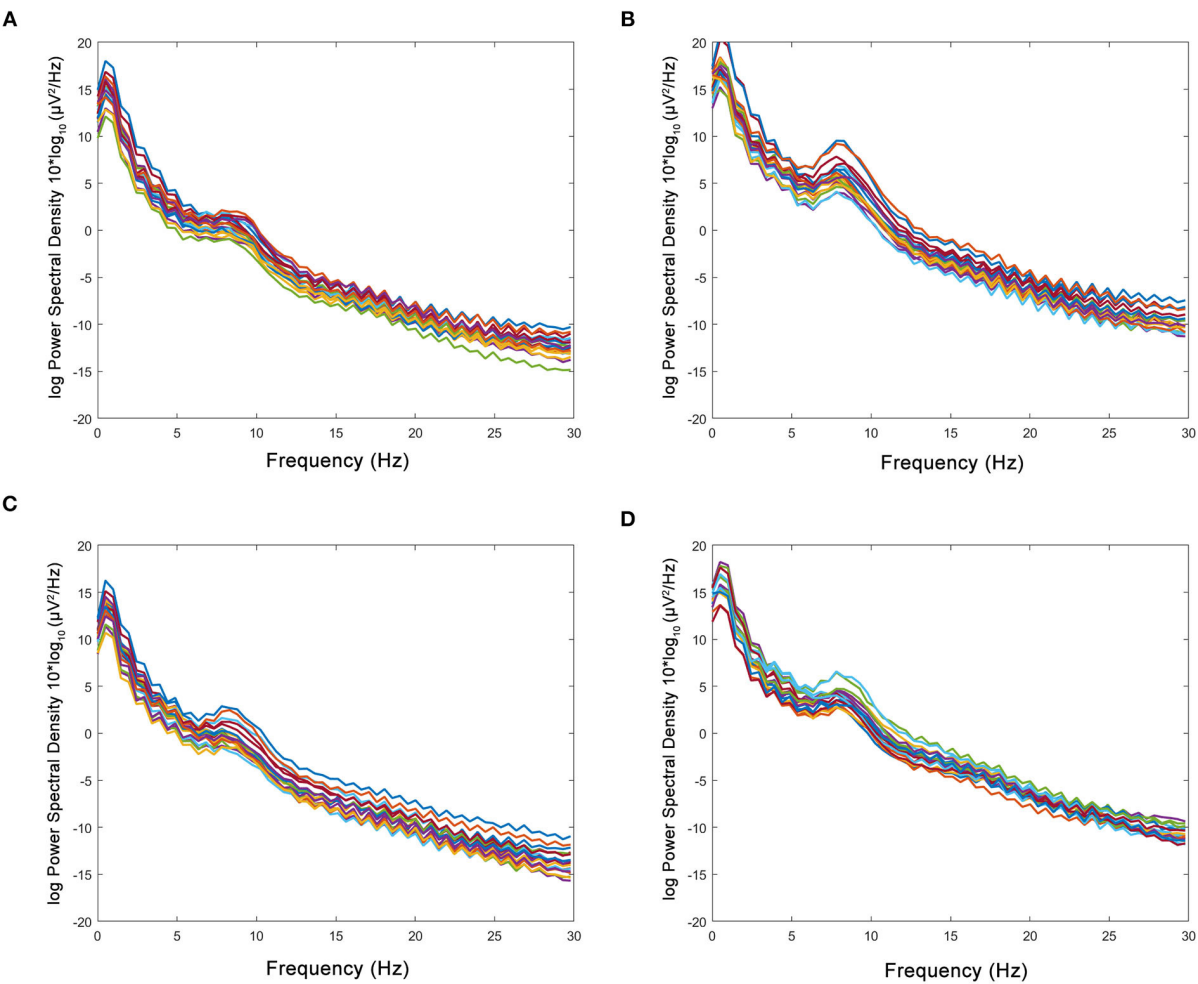
Comparison of power spectral density in the HBO group and the control group. **(A,B)** Power spectral density before and after the treatment in the HBO group. **(C,D)** Power spectral density before and after the treatment in the control group. The HBO group had higher relative power of the α band (16.83 ± 8.60 vs. 10.03 ± 2.84%, *p* < 0.0001) and lower relative power of the δ band (64.47 ± 11.75 vs. 71.05 ± 6.73%, *p* < 0.0001) than the control group.

**Figure 7:**
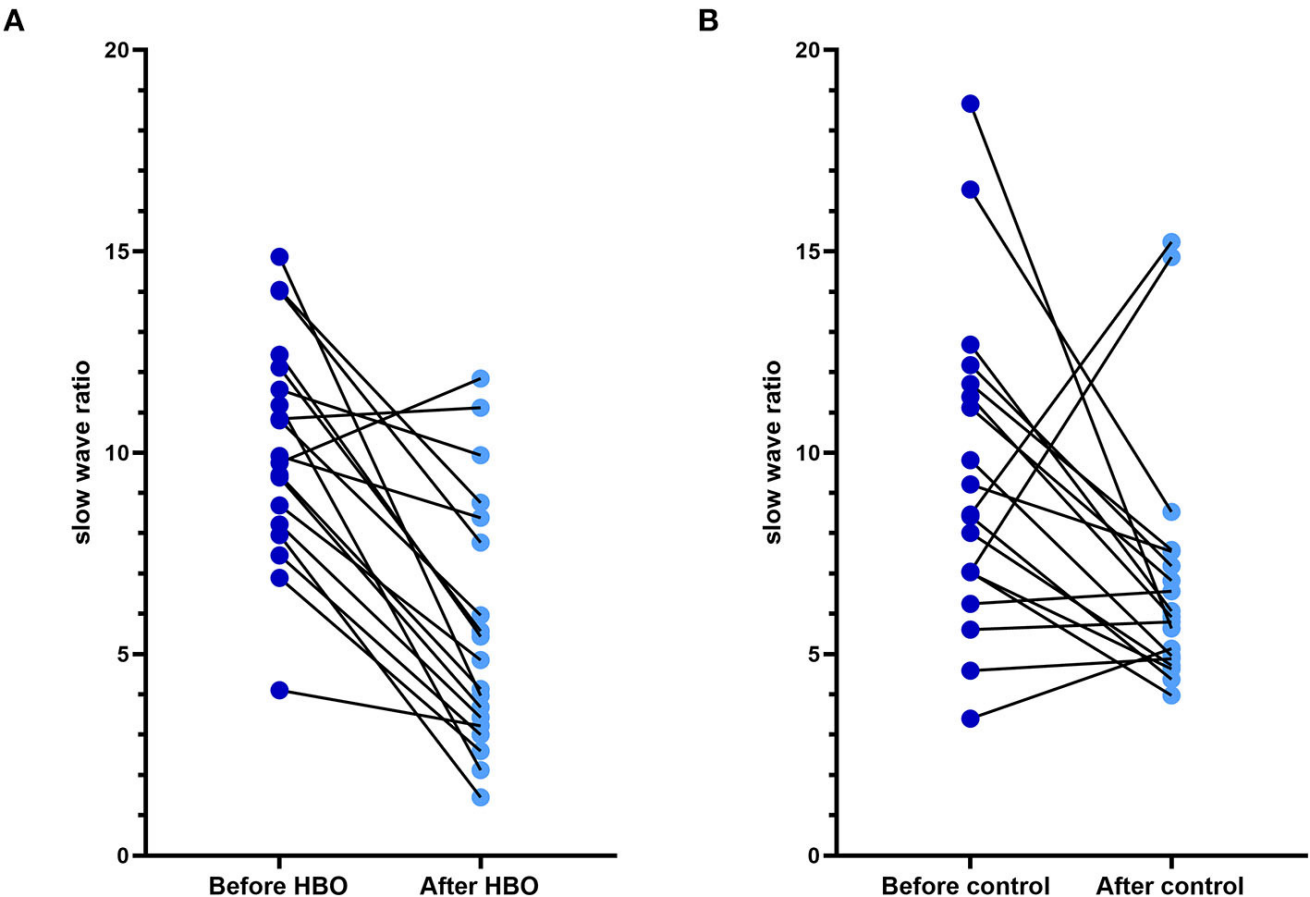
Comparison of the DTABR in the HBO group and the control group. **(A)** DTABRs of different channels in the HBO group before the treatment and 20 days after the treatment; all channels decreased except Fp2 and T4. **(B)** DTABRs of different channels in the control group before the treatment and 20 days after the treatment; no decrease in channels C3, P3, P4, Pz, O1, and O2 was observed.

The figure of power spectral density showed that the increased power was mainly in the alpha band in the HBO group, whereas the control group showed no significant increase after treatment (Figure 6). The DTABRs decreased in all channels except Fp2 and T4 in the HBO group, whereas an increase in six channels (C3, P3, P4, Pz, O1, and O2) and decreases in other channels were observed in the control group (Figure 7). These pervasive decreases in slow waves in scalp recorded locations in the HBO group suggested that HBO affects a wide range of brain electric activity.

### Effects of HBO on serum marker expressions

**Figure 8:**
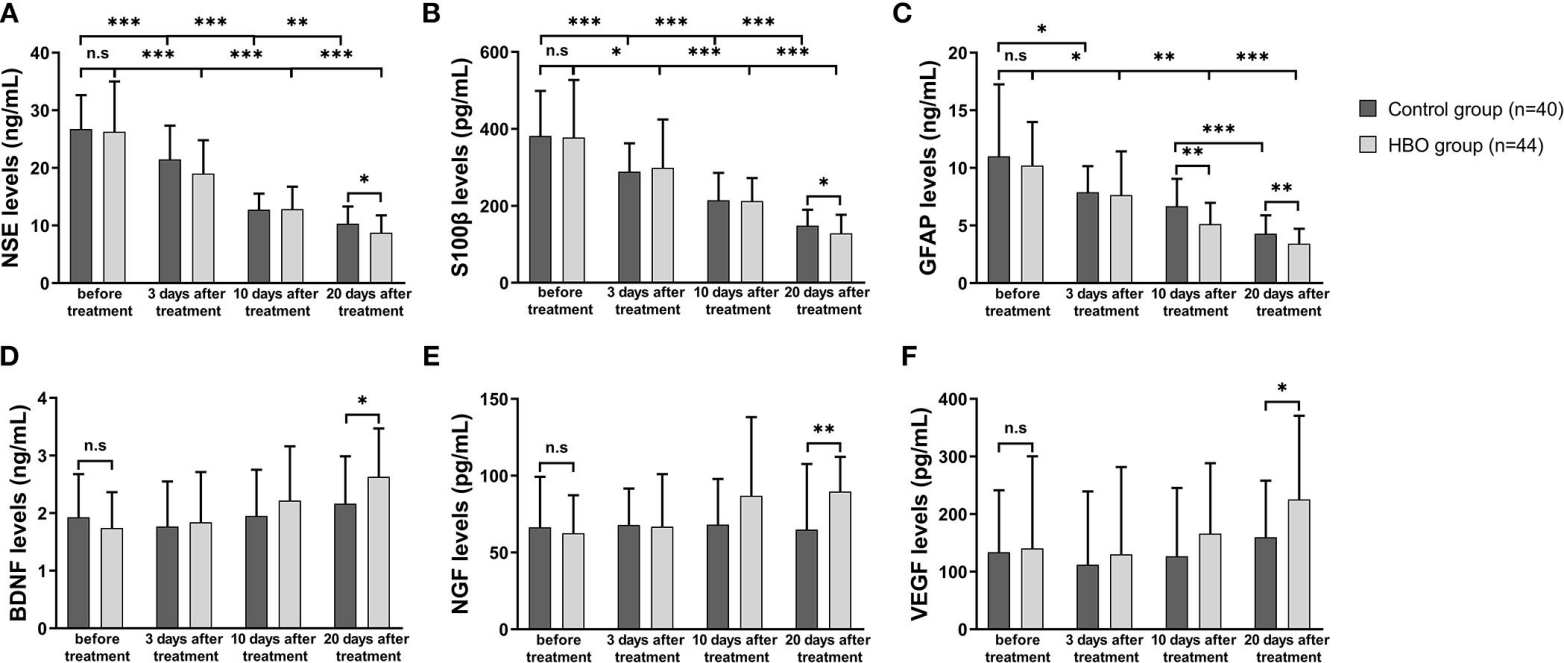
Comparison of serum marker expression before and after the treatment. **(A)** The HBO group had lower NSE expression than the control group at 20 days after the treatment (8.76 ± 3.03 vs. 10.31 ± 3.01 ng/ml, *p* = 0.023). **(B)** The HBO group had lower S100β expression than the control group at 20 days after the treatment (128.57 ± 48.41 vs. 148.50 ± 41.67 pg/ml, *p* = 0.047). **(C)** The HBO group had lower GFAP expression than the control group at 10 days (5.13 ± 1.85 vs. 6.67 ± 2.39 ng/ml, *p* = 0.001) and 20 days after the treatment (3.42 ± 1.30 vs. 4.29 ± 1.61 ng/ml, *p* = 0.008). **(D)** The HBO group had higher BDNF expression than the control group at 20 days after the treatment (2.63 ± 0.84 vs. 2.14 ± 0.84 ng/ml, *p* = 0.009). **(E)** The HBO group had higher NGF expression than the control group at 20 days after the treatment (89.74 ± 22.63 vs. 66.05 ± 41.98 pg/ml, *p* = 0.002). **(F)** The HBO group had higher VEGF expression than the control group at 20 days after the treatment (225.52 ± 145.28 vs. 162.81 ± 99.44 pg/ml, *p* =0.033). *p < 0.05; **p < 0.01; ***p < 0.001.

In both groups, the NSE, S100β and GFAP expression at 10 and 20 days after the treatment was lower than that before the treatment. At 20 days after the treatment, the serum expression of NSE, S100β, and GFAP was significantly lower in the HBO group than the control group (Figures 8A–C), thus indicating that HBO alleviated cell damage and inflammation in neurons and astrocytes. In the HBO group, BDNF, NGF, and VEGF expression at 20 days after the treatment was higher than that before the treatment. At 20 days after the treatment, the serum expression of BDNF, NGF, and VEGF was significantly higher in the HBO group than the control group (Figures 8D–F), thus indicating that HBO promoted neurological function recovery through modulating levels of neurotrophic factors.

### Effect of HBO on prognosis

As shown in Table 3, 6 months after discharge, the HBO group had lower values than the control group in the DRS total score, eye opening, communication ability, motor response, cognitive ability for self-care activities and the functional level. However, the employability between the groups showed no statistical difference (*p* = 0.226). In addition, the HBO group had higher FIM total scores than the control group (*p* = 0.046), and the FIM cognition scores also showed significant differences (*p* = 0.003). Moreover, the HBO group had higher GOSE scores than the control group (*p* = 0.018). In summary, the prognosis at 6 months after discharge was significantly improved by HBO.

**Table 3:** Outcomes evaluated 6 months after discharge through DRS, FIM and GOSE.

	**HBO group** **(*n* = 44)**	**Control group** **(*n* = 36)**	**t**	***P***
DRS	7.82 ± 3.72	14.83 ± 7.94	−4.880	<0.001^*^
Eye opening	0.05 ± 0.30	0.56 ± 1.21	−2.476	0.018^*^
Communication ability	0.48 ± 0.66	1.67 ± 1.67	−4.014	<0.001^*^
Motor response	0.36 ± 0.57	1.58 ± 2.77	−2.596	0.013^*^
Cognitive ability	2.02 ± 1.70	4.81 ± 2.96	−4.996	<0.001^*^
Functional level	2.50 ± 0.98	3.31 ± 1.14	−3.348	0.001^*^
Employability	2.45 ± 0.70	2.64 ± 0.64	−1.221	0.226
FIM	95.27 ± 20.86	80.67 ± 38.39	2.048	0.046^*^
FIM–motor	69.25 ± 17.00	60.81 ± 28.77	1.553	0.126
FIM–cognition	26.02 ± 5.51	20.14 ± 10.30	3.086	0.002^*^
GOSE	4.48 ± 1.42	3.67 ± 1.57	2.422	0.018^*^

HBO, hyperbaric oxygen; DRS, disability rating scale; FIM, functional independence measure; GOSE, Glasgow outcome scale–extended.

* P < 0.05.

## Discussion

HBO therapy has been widely used in the treatment of craniocerebral injury in China, and the Consensus of Chinese experts on severe neurological rehabilitation has proposed that HBO therapy is a major wake-up treatment method in 2017 (21). The therapeutic parameters of hyperbaric oxygen therapy for traumatic brain injury have not been unified, and treatment pressure is in the range of 1.5 to 2.5 ATA worldwide. The application of HBOT for moderate and severe TBIs is usual from 1.5 to 2.0 ATA according to the expert consensus of hyperbaric oxygen for craniocerebral trauma in China (2021 edition) (22). 2.0 ATA was generally accepted by clinical doctors for a balance of clinical efficacy and safety, and there were no patients with barotrauma or other side effects that were exposed to environmental pressure 2.0 ATA in our study. A meta-analysis has shown that HBO treatment increases GCS scores in patients with mild and severe TBI, and decreases the overall mortality (23). CRS-R scores were usually used to assess recovery of consciousness following TBI and evaluate the efficacy of various therapeutic interventions on disorders of consciousness (24–26). In the present study, the increases in CRS-R scores in patients with HBO treatment demonstrated that this modality improves the recovery of consciousness after TBI.

The cognitive impairment that occurs after TBI is more severe and long-lasting than other impairments, mainly manifesting as poor concentration, memory loss, and diminished executive ability (27). In this study, the patients with TBI were unable to cooperate in completing the mini mental state examination (MMSE) and the Montreal cognitive assessment (MoCA) for reasons including tracheotomy, coma, oral expression disorder, and irritability. Consequently, the RLAS-R was selected, which is more acceptable to patients, and describes the cognitive and behavioral patterns of recovery in patients with TBI after injury, considering the patients' state of consciousness and the assistance that they require to perform cognitive and physical functions (28). A previous study has demonstrated that, in patients with TBI and chronic neurological injury, HBO promotes the recovery from neurocognitive impairment, particularly memory impairment, attention deficit, and executive dysfunction by inducing neuroplasticity, inducing cerebral angiogenesis and improving the structural disruption associated with cognition (20). Investigators (29) have used single photon emission computed tomography to evaluate cerebral blood flow and have found that HBO ameliorates cognitive deficits in patients with TBI at all severity levels, through altering brain perfusion in the anterior cingulate and the postcentral cortex, in the pre-frontal and temporal areas. In this study, continual HBO therapy ameliorated cognitive impairment in patients with moderate to severe TBI, and the efficacy at 20 days after HBO was better than that at 10 days after HBO treatment. Moreover, 6 months after discharge, the HBO group had better cognitive function than the control group, according to DRS and FIM scales, which indicated that HBO ameliorated not only cognitive impairment in the short term but also cognitive impairment and prognosis of patients with TBI in the long term.

The Stockholm CT score is usually used to grade severity and predict the prognosis of TBI (30), thus rapidly and accurately reflecting the state of injury and enabling dynamic monitoring of the development and prognosis of structural lesions. This study used the Stockholm CT score to compare intracranial injuries before and after treatment; HBO decreased the intracranial hematoma volume after TBI, probably through increasing oxygen concentrations and promoting circulation. However, small lesions in the brainstem and the cerebellum may be missed using CT scanning. Combined neuroimaging with multiple examination methods could be helpful in dynamically evaluating the clinical efficacy of HBO in patients with TBI.

EEG has been applied for detection of cortical function in patients with traumatic DoC and the prediction of prognosis and differential diagnosis in patients with TBI and disorders of DoC (31–33). The clinical utility of QEEG has been demonstrated for electrical abnormalities and network dysfunction that includes an elevation of frontal/temporal δ and θ powers as well as abnormalities in functional connectivity (34). Some QEEG parameters with potential in outcome prediction were found, and α power and variability of the relative fast θ power were reported as the best QEEG parameters for outcome prediction of severe TBIs (35). EEG δ wave power ratios (δ-to-α ratios, DAR; δ-to-θ ratios, DTR; and DTABR) and relative power (RP) were effective biomarkers of neurological changes due to TBI and stroke (36, 37). In addition, increased δ and reduced α activity in the brain areas are associated with an increase in the severity of confusion following TBI, and DAR is a marker of post-traumatic confusional state and functional recovery post-injury (37). In this study, QEEG was used to evaluate the therapeutic effects of HBO on patients with TBI after HBO, the relative power of the δ band decreased, and that of the α band increased, and the DTABRs decreased in most observed channels. HBO might have a positive role in awakening through reducing extensive slow waves due to TBI.

Several blood biomarkers are used to assess injury severity and outcomes in TBI, and they are expected to become targets of treatment (38, 39). S100β, a monitoring marker of ongoing injury in adults and a surrogate marker for treatment efficacy, plays an important role in brain injury (40). Furthermore, GFAP and NSE are released after injury and have also been demonstrated to be associated with outcomes in patients with TBI (41). NSE, S100β, and GFAP are considered to negatively correlate with TBI severity and prognosis, and to represent cognitive deficits (42–44). Our study has found that HBO partially alleviates secondary injury *via* downregulating the expression of NSE, S100β, and GFAP. BDNF, NGF, and VEGF are considered useful methods to evaluate recovery from neurological injury and vascular damage (45, 46). BDNF has been found to positively correlate with outcomes after TBI (47), and NGF and VEGF have been associated with neuronal survival and fracture healing (48). Furthermore, VEGF plays an important role in angiogenesis and has been demonstrated to increase after HBO in TBI rats (49). BDNF, NGF, and VEGF increased to varying degrees after 20 sessions of HBO therapy in this study, thereby potentially indicating faster recovery and improved prognosis after HBO therapy. Thus, these results suggested that HBO may alleviate secondary injury in TBI *via* modulating biomarkers associated with neuronal injury, astrocyte injury, and neurotrophic action.

HBO therapy has shown positive effects on patients with TBI by decreasing hospital stay, disability, and mortality, and improving social behavior and prognosis (8, 9, 50). A recent study has reported that HBO combined with rehabilitation training improves activities of daily living, movement ability, and cognitive function in patients after TBI (19). In our study, the DRS, FIM, and GOSE score at 6 months after discharge revealed that the patients receiving HBO therapy had better outcomes than controls in terms of cognition, communication ability, and the functional level, except employability. This study not only demonstrated the efficacy of HBO therapy in DoC, cognitive impairment in patients with TBI in the short term, but also indicated that HBO improves the prognosis of patients with TBI in the long term.

## Study limitations

The study has several limitations. First, this study focused on 20 consecutive days of HBO, because of various conditions and restrictions, such as hospitalization periods, personal financial differences, and management of the oxygen chamber. Second, the study had a small sample size. Finally, proton magnetic resonance spectroscopy and diffusion tensor imaging were used to assess the clinical curative effects of HBO therapy. However, many patients with TBI were unable to cooperatively complete the examination, owing to severe injury, irritability, and other reasons; thus, only five patients' data were collected and were insufficient for statistical analysis.

## Conclusions

In conclusion, HBO improves consciousness, cognitive function, and prognosis in patients with severe or moderate TBI through decreasing TBI-induced hematoma volumes, promoting the recovery of EEG rhythms, and modulating the expression of serum NSE, S100β, GFAP, BDNF, NGF, and VEGF.

## Data availability statement

The original contributions presented in the study are included in the article/supplementary material, further inquiries can be directed to the corresponding author.

## Ethics statement

The studies involving human participants were reviewed and approved by the Medical Ethics Committee of the Affiliated Hospital of Nantong University (Approval No. 2020-K029). The patients/participants provided their written informed consent to participate in this study.

## Author contributions

SL contributed to the conception, design, and study supervision. WY and LW contributed to the analysis and interpretation of data. YC and YJ drafted the article. SL, FH, LS, and SW critically revised the article. YC contributed to the statistical analysis. YC, LW, WY, FH, YJ, LS, SW, and SL contributed to the acquisition of data and reviewed submitted version of the manuscript. All authors contributed to the article and approved the submitted version.

## Funding

This project was funded by National Natural Science Foundation of China (No. 81702223).

## Conflict of interest

The authors declare that the research was conducted in the absence of any commercial or financial relationships that could be construed as a potential conflict of interest.

## Publisher's note

All claims expressed in this article are solely those of the authors and do not necessarily represent those of their affiliated organizations, or those of the publisher, the editors and the reviewers. Any product that may be evaluated in this article, or claim that may be made by its manufacturer, is not guaranteed or endorsed by the publisher.
